# Supplementary material for: Genotoxic stress triggers the activation of IRE1α-dependent RNA decay to modulate the DNA damage response
Source: Nat Commun. 2020 May 14;11:2401. doi: 10.1038/s41467-020-15694-y (PMC7224204; doi:10.1038/s41467-020-15694-y)
Supplement: Supplementary file 1 — Supplementary Information [file 41467_2020_15694_MOESM1_ESM.pdf]

**Supplementary Information**

# Genotoxic stress triggers the selective activation of IRE1α-dependent RNA decay to modulate the DNA damage response.

*Dufey et al*., 2020 Nature Communications

## Supplementary figure 1

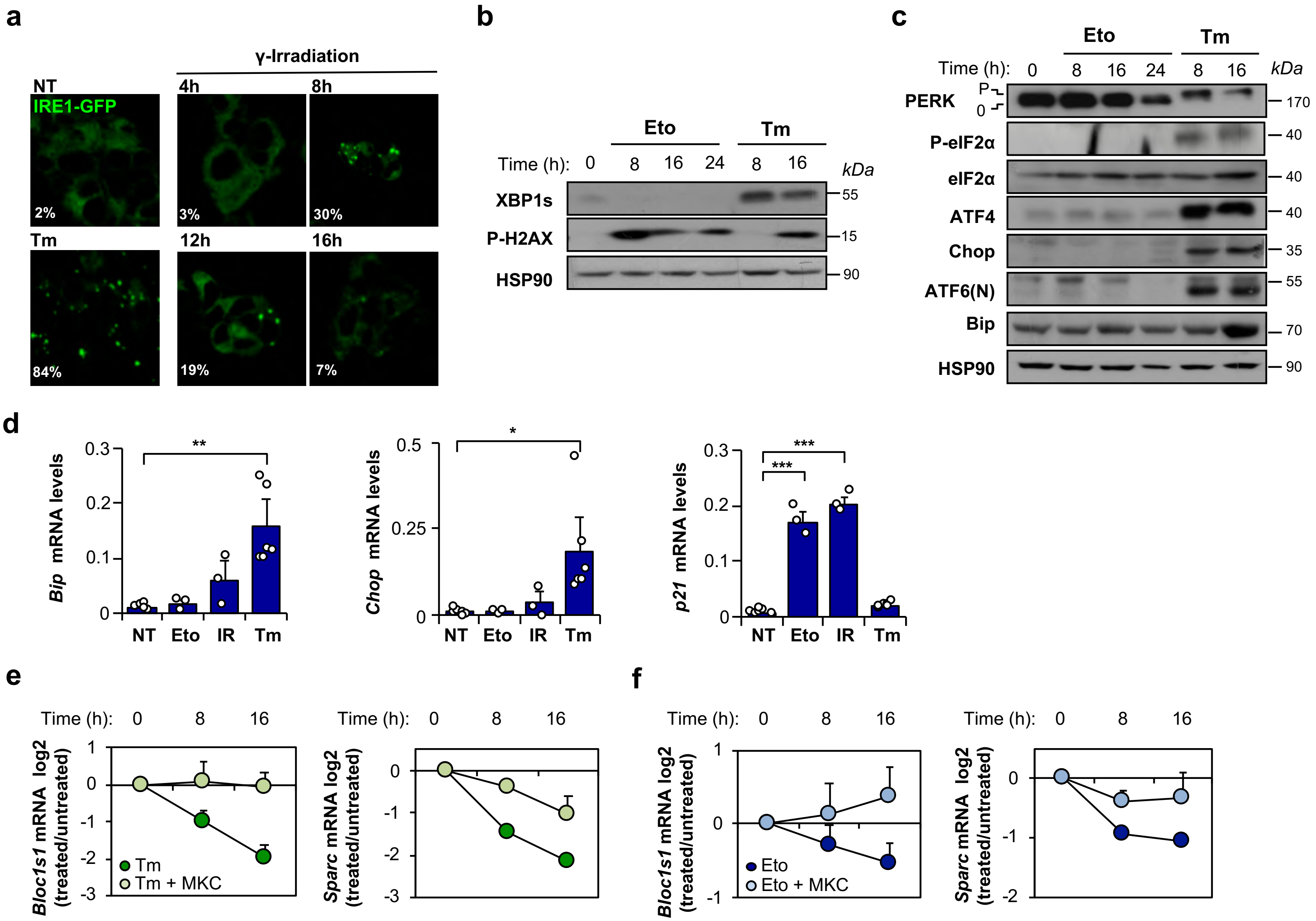

**Supplementary Figure 1. DNA damage triggers the selective activation of IRE1α-dependent RNA decay in the absence of an ER stress signature. a** TREX IRE1-3F6H-GFP cells were exposed to 25 Gγ of ionizing radiation (IR), and then fixed at indicated time points post-IR to quantify the percentage of cells containing IRE1α-GFP foci (bottom left numbers). Treatment with 500 ng/mL tunicamycin (Tm) for 4h was used as positive control. **b** WT MEF cells were treated with 10 μM etoposide (Eto) at indicated time points and the expression levels of XBP1s and phosphorylated H2AX (P-H2AX) detected by western blot. Hsp90 was monitored as loading control. Treatment with 500 ng/mL Tm was used as positive control. **c** WT MEF cells were treated with 10 μM Eto or 500 ng/ml Tm at indicated time points. Then, the expression levels of indicated ER stress proteins monitored by western blot. Hsp90 was monitored as loading control. **d** *HSPA5 (BiP), DDIT3 (Chop)* and *CDKN1A (p21*) mRNA were quantified by real-time PCR in cells treated with to 25 Gγ of IR, 10 μM Eto or 500 ng/mL Tm for 16 h (n = 3-6). **e** and **f** WT MEF cells were treated with 10 μM Eto, 500 ng/mL of Tm, or in combination with the IRE1α RNase inhibitor 25 μM MKC-8866 for 16h. *Bloc1s1* and *Sparc* mRNA levels were monitored by real-time PCR and normalized with the expression levels of *Rpl19* mRNA (n = 4). In all panels, data is shown as mean ± s.e.m.; *$p < 0.05$, **$p < 0.01$ and ***$p < 0.001$, based on **(d)** *One-way ANOVA followed Tukey's test. Data is provided as a Source Data file.*

## Supplementary Figure 2

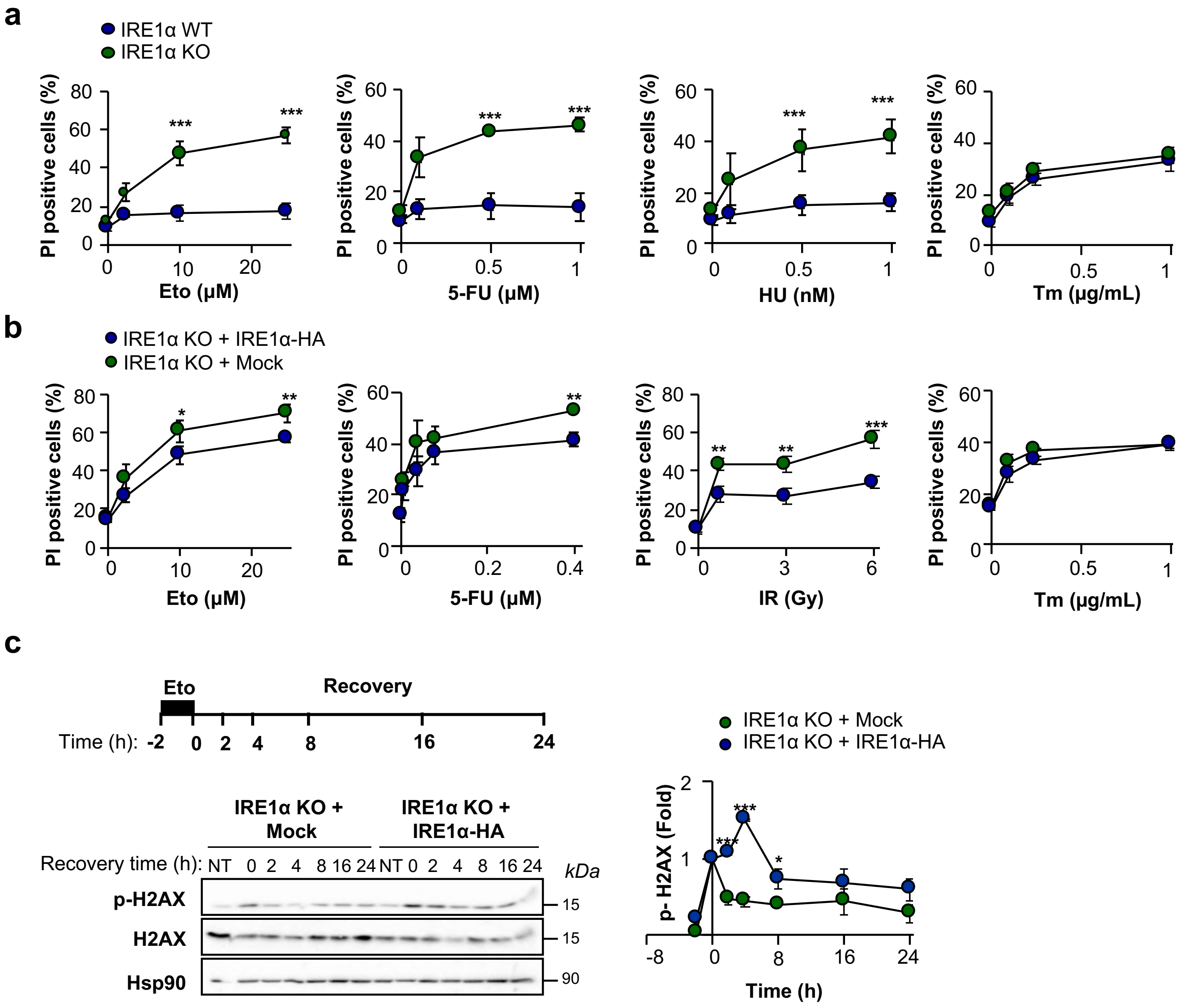

**Supplementary Figure 2. IRE1α deficiency reduces the survival of cells under DNA damage and alters the phosphorylation of the histone H2AX. a** WT and IRE1α KO MEF cells were treated with indicated concentration of etoposide (Eto), 5-fluorouracilo (5-FU), hidroxyurea (HU) and tunicamycin (Tm). After 24h, cell viability was analyzed by propidium iodide (PI) staining and FACS analysis (n = 3). **b** IRE1α KO (Mock) and reconstituted cells with an IRE1α-HA expression vector were treated with indicated concentrations of Eto, 5-FU, gama irradiation (Gy), or Tm. After 24h cell viability was analyzed after PI staining and FACS analysis (n = 3). **c** Diagram of the experimental setting (upper panel). IRE1α KO (Mock) and IRE1α-HA reconstituted cells were pre-incubated with 1 μM Eto for 2 h and washed three times with PBS and fresh cell culture media was added. The decay of phosphorylated H2AX (P-H2AX) was monitored over time by western blot (middle panel). Quantification of the levels of P-H2AX in cells stimulated with Eto (bottom panel). Hsp90 was monitored as loading control (n = 3). In all panels, data is shown as mean ± s.e.m.; *$p < 0.05$, **$p < 0.01$ and ***$p < 0.001$, based on (**a,b,c)** *two-way ANOVA followed Bonferroni's test. Data is provided as a Source Data file.*

## Supplementary Figure 3

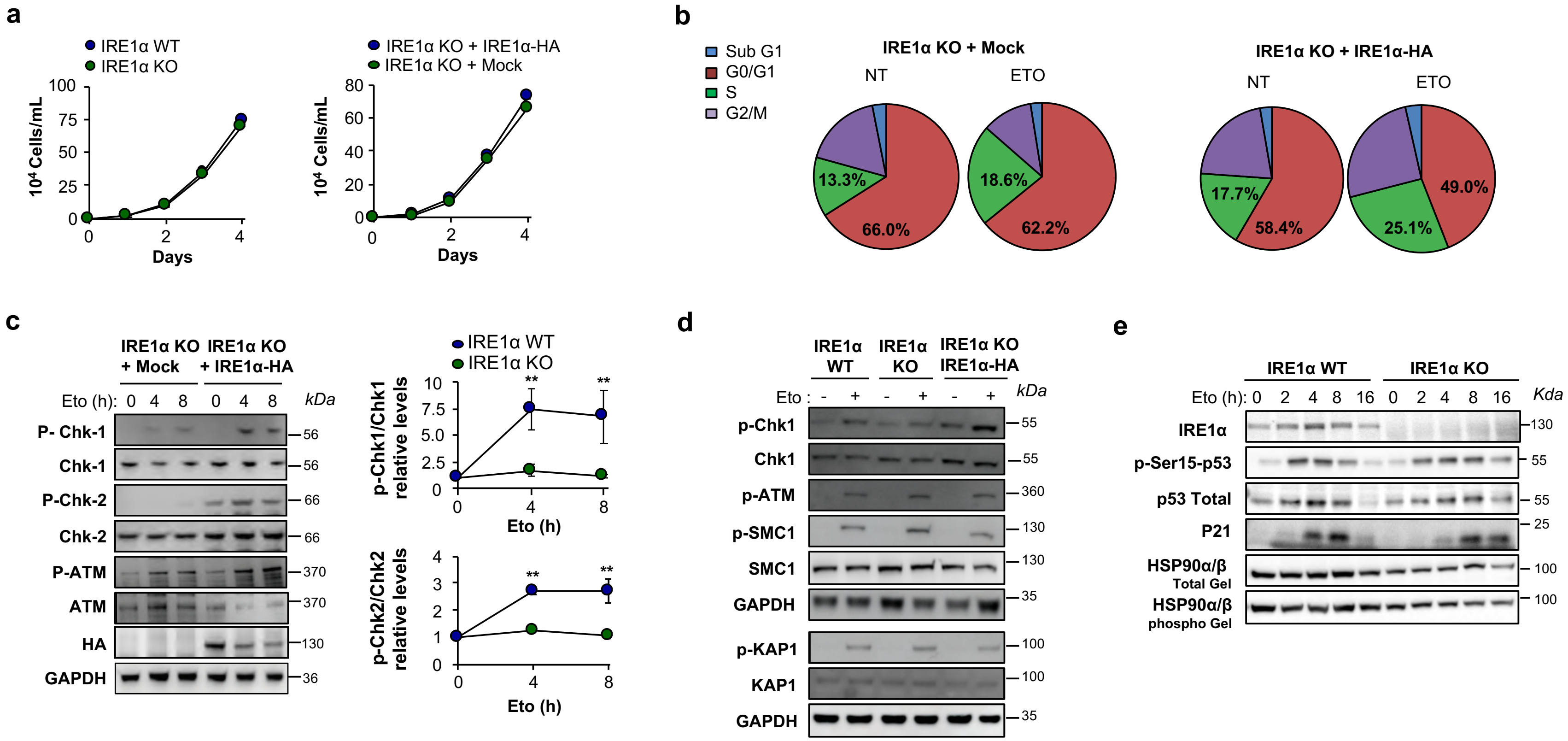


**Supplementary Figure 3. IRE1α deficiency impairs the DNA damage response. a** Cell proliferation was monitored in WT and IRE1α KO MEF cells using trypan blue exclusion staining (left panel). In addition, IRE1α KO (Mock) and reconstituted cells with an IRE1α-HA expression vector were analyzed (n = 3). **b** IRE1α KO (Mock) and reconstituted cells with an IRE1α-HA were treated with 10 μM etoposide (Eto) for 8h and cell cycle was analyzed by propidium iodide (PI) staining. Quantification of the percentage of cells in G0/G1 and S phases is shown. **c** IRE1α KO (Mock) and reconstituted cells with an IRE1α-HA were treated with 10 μM Eto at indicated time points. An expression level of indicated proteins was monitored by western blot analysis (left panel) Quantification of CHK1 and CHK2 phosphorylation was performed (right panel) (n = 3). **d** MEF WT**,** IRE1α KO (Mock) and reconstituted cells with an IRE1α-HA

were treated with 10 μM Eto (8h). An expression level of indicated proteins was monitored by western blot analysis. **e** WT and IRE1α KO MEF cells were treated with 10 μM Eto at indicated time points. An expression level of indicated proteins was monitored by western blot analysis. In all panels, data is shown as mean ± s.e.m.; *$p < 0.05$, **$p < 0.01$ and ***$p < 0.001$, based on (**a,c**) *two-way ANOVA followed Bonferroni's test. Data is provided as a Source Data file.*

## Supplementary Figure 4

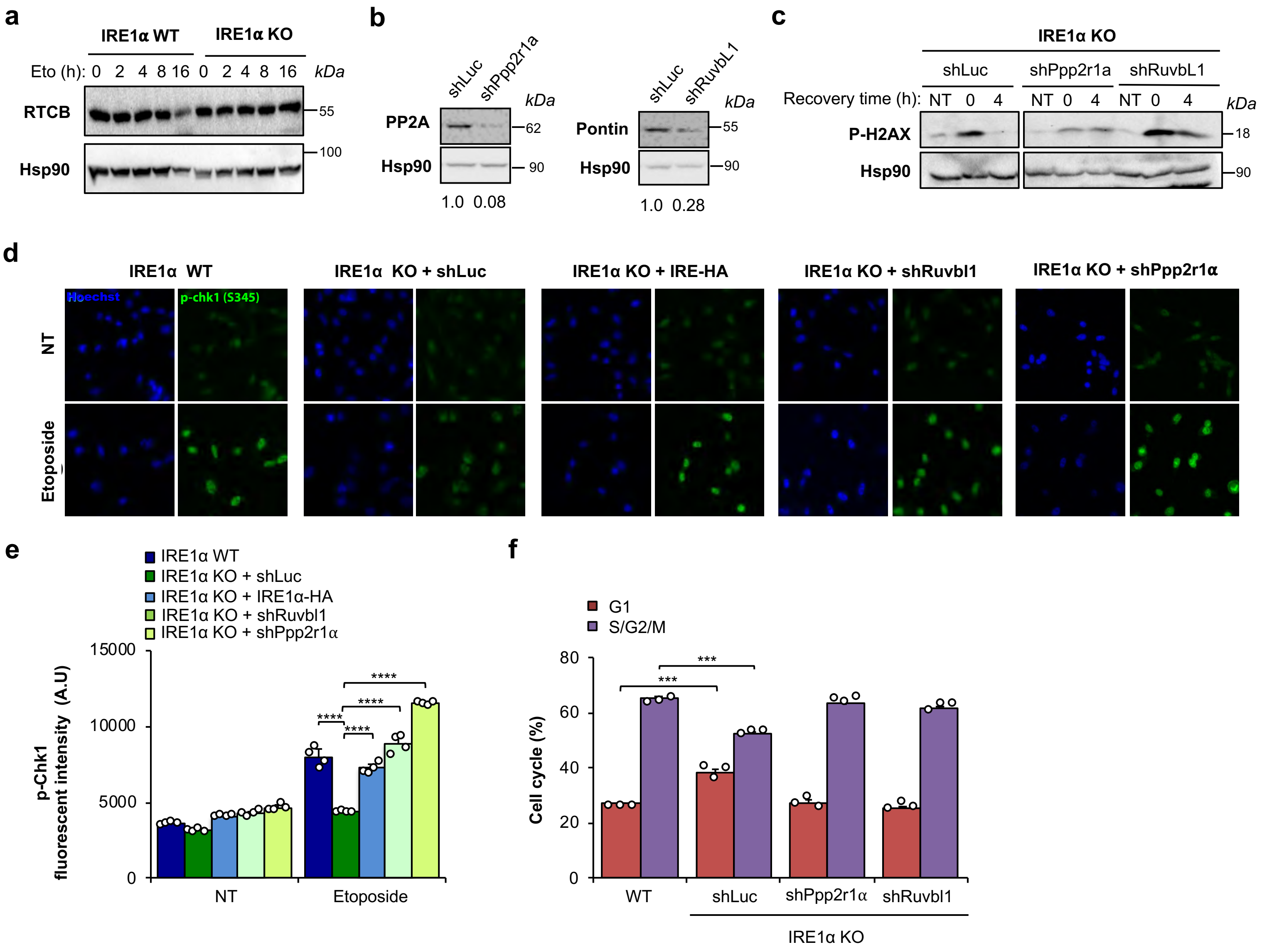

**Supplementary Figure 4. Knocking down *Ppp2r1a* or *Ruvbl1* in IRE1α KO cells augmented the phosphorylation of H2AX under DNA damage. a** WT and IRE1α KO MEF cells were treated with 10 μM Etoposide (Eto) at indicated time points. An expression level of RTCB was monitored by western blot analysis. **b** IRE1α KO cells were stably transduced with shRNA constructs to target the *Ppp2r1a*, *Ruvbl1* or Luciferase (Luc) mRNA. Expression levels of PP2A and Pontin were determined by Western blot analysis to the indicated proteins. Hsp90 was monitored as loading control. **c** IRE1α KO MEF cells were stably transduced with lentiviruses expressing shRNAs against the *Ppp2r1a* (*shPpp2r1a*), *RuvbL1* (*shRuvbL1*) or luciferase as control (*shLuc*). Cells were incubated with 1 μM Eto for 4h. Then, cells were washed three times with PBS and fresh culture media was added. The expression levels of phosphorylated-H2AX were monitored by western blot. Hsp90 was monitored as loading control. **d-e** MEF WT, MEF IRE1α KO reconstituted cells with an IRE1α-HA and IRE1α KO MEF cells stably transduced with lentiviruses expressing shRNAs against the *Ppp2r1a* (shPpp2r1a), *RuvbL1* (shRuvbL1) or luciferase as control (shLuc), were treated 8 hours with 10 μM Eto. Then immunofluorescence analysis was performed to detect the CHK1 phosphorylation. The results were graphed as arbitrary units (A.U) of green intensity (n = 4). **f** MEF WT and IRE1α KO MEF cells stably transduced with lentiviruses expressing shRNAs against the *Ppp2r1a* (shPpp2r1a), *RuvbL1* (shRuvbL1) or luciferase as control (shLuc) were treated with 10 μM Eto and cell cycle was analyzed by propidium iodide (PI) staining. Quantification of the percentage of cells in G1 and S/G2/M phases is shown (n=3). Representative images of three independent experiments. In all panels, data is shown as the mean ± s.e.m.; *$p < 0.05$, **$p < 0.01$ and ***$p < 0.001$, based on (**e,f)** *two-way ANOVA followed Bonferroni's test. Data is provided as a Source Data file.*

## Supplementary Figure 5

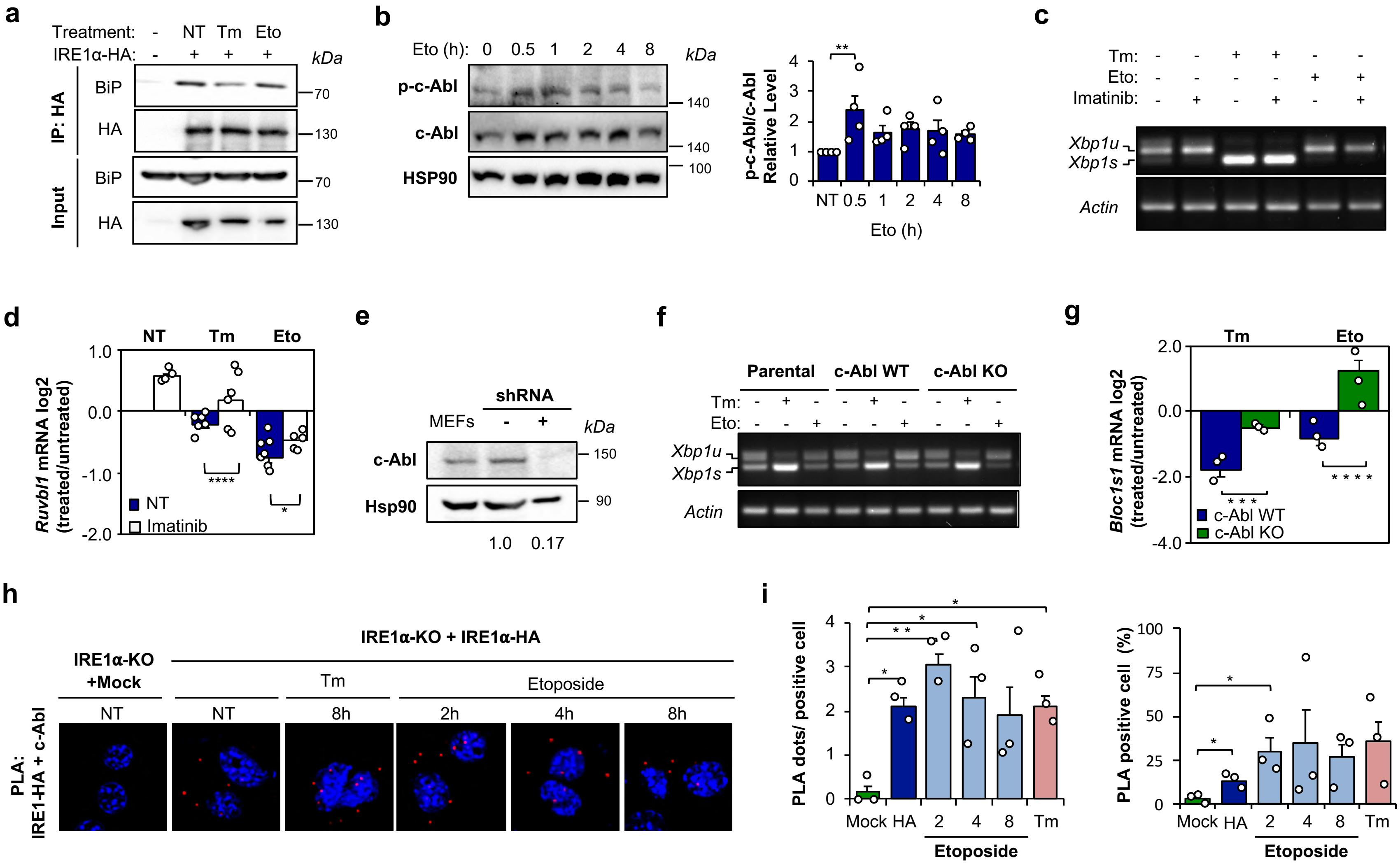

**Supplementary Figure 5. ER stress and DNA damage synergize to induce RIDD without affecting XBP1 mRNA splicing. a** IRE1α KO cells were reconstituted with IRE1α-HA and treated with 500 ng/ml tunicamycin (Tm) for 4 h or 10 μM etoposide (Eto) for 16h then protein extract were prepared in 1% NP-40 buffer. IRE1α-HA was immunoprecipitated (IP) and analyzed by western blot the presence of BiP in the isolated protein complexes. **b** c-Abl phosphorylation was monitored in WT MEFs cells treated with 10 μM Eto at indicated time points followed by western blot analysis (n = 4). **c** IRE1α WT MEF cells were treated with 10 μM Eto, 500 ng/mL tunicamycin (Tm) or in combination with c-Abl inhibitor Imatinib 10 μM for 8h, to measuring the *Xbp1* mRNA splicing by PCR. **d** WT MEF cells were treated with 10 μM Eto, 500 ng/mL of Tm, or in combination with the c-Abl inhibitor Imatinib 10 μM for 8 h. *Ruvbl1* mRNA levels were monitored by real-time PCR (n = 4-7). **e** MEFs cells were transfected with shRNA scramble or shRNA against *c-Abl*, and through WB we measured the protein knockdown, using Hsp90 as loading control. **f** MEF WT (Parental), MEF CRISPR-Control and MEF CRISPR-Abl cells were treated with 10 μM Eto, 500 ng/mL Tm for 8h, to measuring the Xbp1 mRNA splicing by PCR. **g** MEF CRISPR-Control and MEF CRISPR-Abl cells were treated with 10 μM Eto or 500 ng/mL of Tm for 8h. *Bloc1s1* mRNA levels were monitored by real-time PCR and normalized with the expression levels of *Rpl19* mRNA (n=3). **h-i** IRE1α KO (Mock) and reconstituted cells with an IRE1α-HA (HA) were treated with 10 μM Eto at indicated time points and stained with a proximity ligation assay (PLA) using an anti-HA or anti-c-Abl antibodies and analyzed by confocal microscopy. Left panel: Number of dots per cell analyzed and percentage of PLA positive cells were quantified (n = 3). In all panels, data is shown as mean ± s.e.m.; $*p < 0.05$, $**p < 0.01$ and $***p < 0.001$, based on (**b,i)** One-way ANOVA followed Tukey's test, **(d)** two-tailed unpaired t-Student's test, (**g)** *two-way ANOVA followed Bonferroni's test. Data is provided as a Source Data file.*

## Supplementary Figure 6

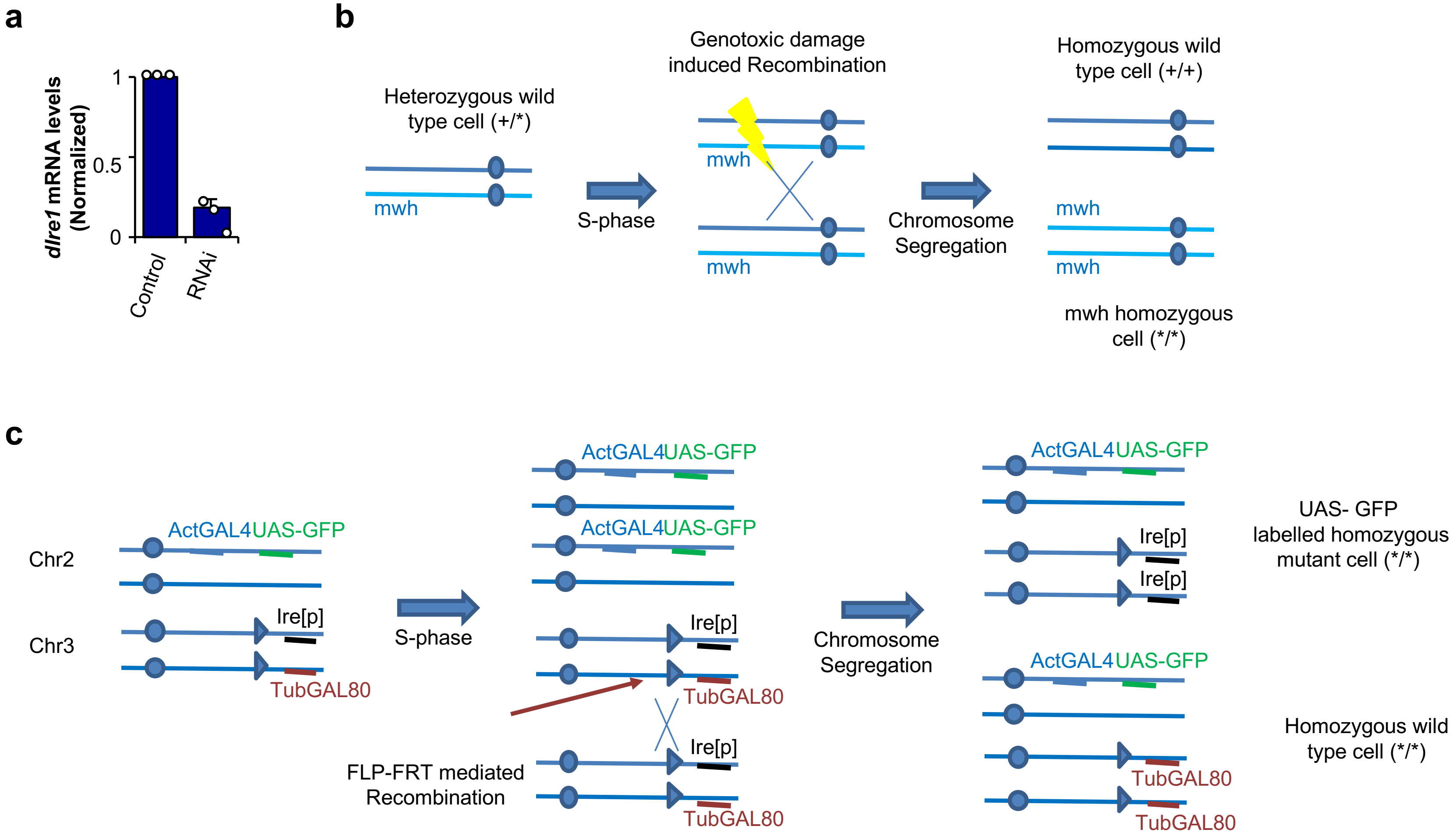


**Supplementary Figure 6. Study of IRE1α function on the DNA damage response in *D. melanogaster*. a** *D. melanogaster* IRE1α homologue (dIre1) was knocked down by expressing a specific RNAi construct under the control of tubulin-Gal4 driver using the UAS-GAL4 system. The levels of dIre1 mRNA were evaluated from control (GFP-IR;;Tub-GAL4) and dIre1 RNAi expressing larvae (Ire-IR;;

Tub-GAL4) by real-time RT-PCR (n = 3). **b** Scheme of the SMART assay. The case of the $mwh^{1}$ mutation is presented, however an equivalent scenario applies for the $flr^{3}$mutation in the homolog chromosome. $mwh^{1}$ and $flr^{3}$ are recessive mutations, therefore heterozygous cells display no phenotype. DNA breaks induced by the genotoxic agents favor mitotic recombination between homolog chromosomes, this generates one cell which receives both wild type alleles (+/+, no phenotype) and the other inherits both mutant alleles (*/*, $mwh^{1}$phenotype). **c** Scheme of the MARCM assay used. The act-GAL4 driver (Actin-Gal4) and the UAS-GFP transgenes are located in the second chromosome. The Tub-GAL80 construct coding for a competitive inhibitor of the GAL4 transcription factor and the FRT80A recombination site are located in left arm of the third chromosome (3L). dIre1 null mutation and the same FRT site are present in the homolog chromosome. FLP induction promotes the recombination between FRT sites in dividing cells. This stochastic event during mitosis generates a descendant inheriting the two Tub-GAL80 alleles, while the other segregates both dIre1mutant alleles. The lack of Tub-GAL80 in dIre1mutant cells allows Gal4 activation of GFP expression. *Source data are provided as a Source Data file.*

## Supplementary Figure 7

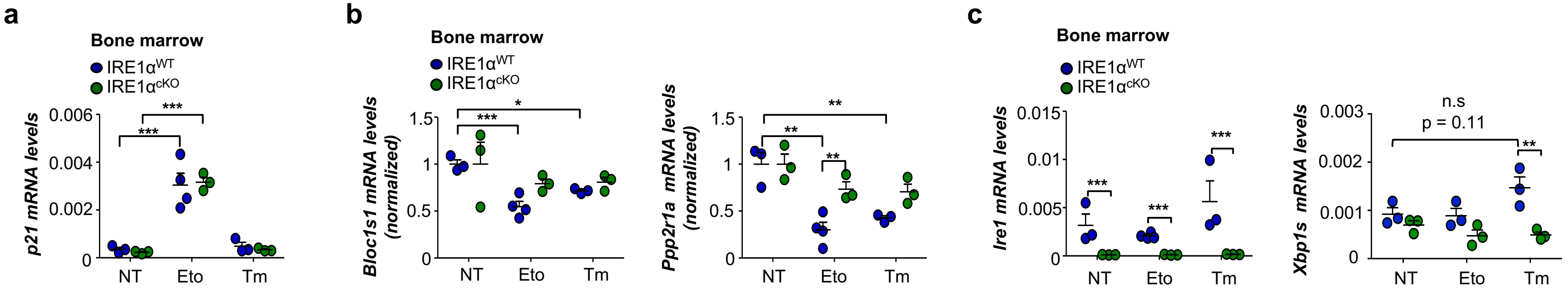


**Supplementary Figure 7. Deletion of IRE1α in bone morrow alters the DDR under genotoxic stress.** Mouse IRE1α was conditionally deleted in the liver and bone morrow using the MxCre and LoxP system (IRE1α$^{cKO}$). Mice were intraperitoneally injected with 50 mg/Kg etoposide (Eto) or 1 mg/Kg tunicamycin (Tm) and sacrificed 6 hours later. **a** Total mRNA levels of *p21* were measured in the bone morrow by real-time PCR and normalized to the expression levels of actin. **b** *Bloc1s1* and *Ppp2r1a* mRNA expression levels were monitored by RT-PCR in samples indicated in a (n = 3-4 mice per group). **c** IRE1α mRNA levels and *Xbp1* mRNA splicing was monitored by RT-PCR in the same samples indicated in a. In all panels, data is shown as the mean ± s.e.m.; *$p$ < 0.05, **$p$ < 0.01 and ***$p$ < 0.001, based on **(a,b,c)** two-way ANOVA followed Bonferroni's test. *Data is provided as a Source Data file.*

# Supplementary Figure 8

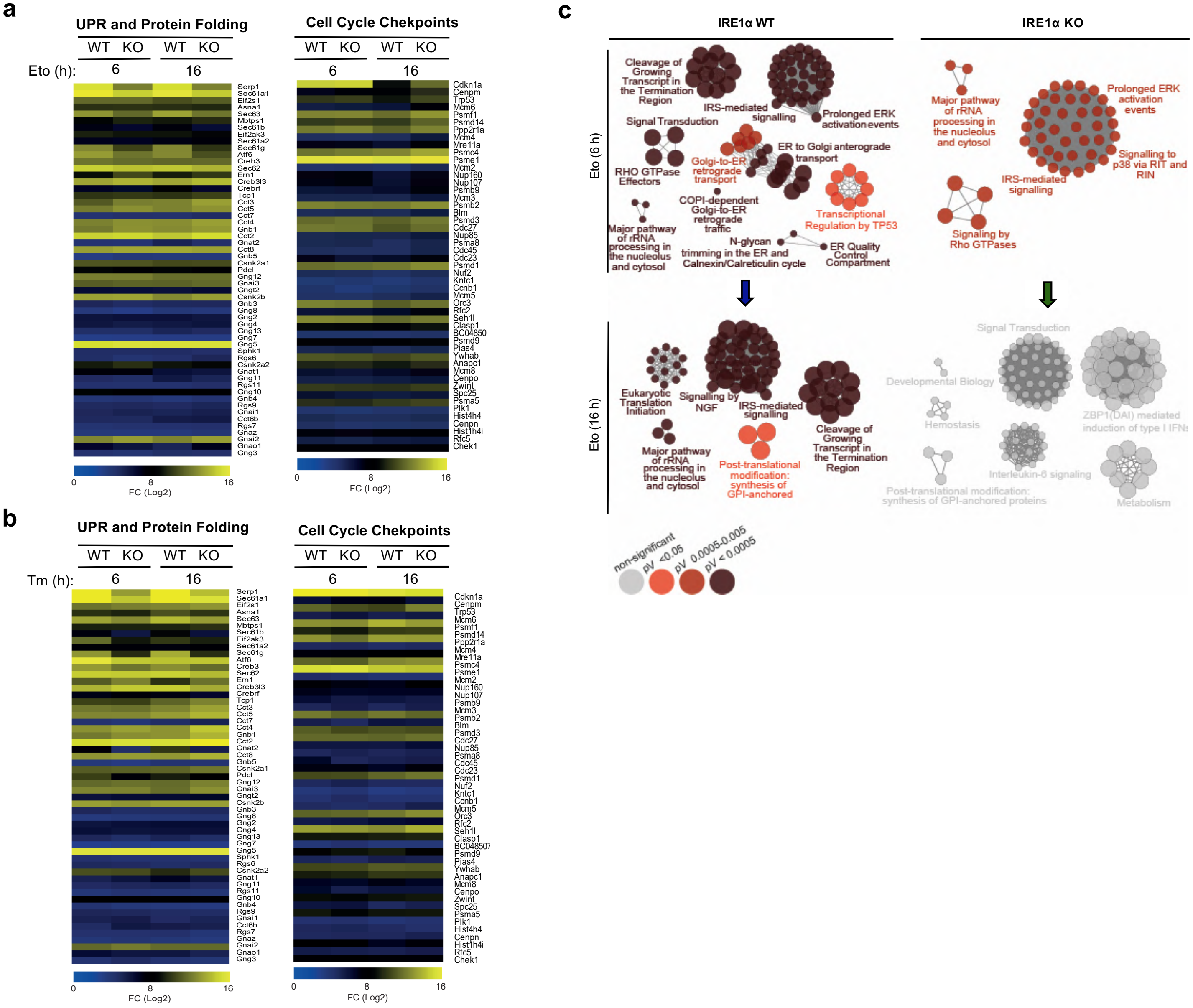

**Supplementary Figure 8. Functional networks altered upon deleting Ern1 in the liver after etoposide treatment.** **a** Heatmap representation of UPR, ER stress signaling (ERS) and apoptosis/DDR related mRNAs derived from WT or IRE1α$c^{KO}$ mice after 6h or 16h of etoposide treatment as described in Figure 6a. **b** Reactome pathway enrichment analysis associated to WT (left panel) and IRE1α$c^{KO}$ (right panel) mice after 6h (upper panel) and 16h (lower panel) of tunicamicyn treatment. Briefly, functional pathway analysis was carried out 12 using ClueGO v2.3.2 plugin in Cytoscape 3.2.0 software, using Reactome (v09.11.2016) enrichment pathway database in default mode. Only pathways with a significant pValue (P<0.05 with Bonferroni correction) where displayed. **c** Heatmap of IRE1α-related gene signature in WT or IRE1α$^{cKO}$ after 6h or 16h of tunicamycin treatments. *Data is provided as a Source Data file.*

**Supplementary Table 1. Primers used in the study**

| mRNA | Forward | Reverse |
|---|---|---|
| PCR | | |
| mXBP1S | AAGAACACGCTTGGGAATGG | CTGCACCTGCTGCGGAC |
| mβ-Actin | ACCACCATGTACCCAGGCA | CTCAGGAGGAGCAATGATCTTGAT |
| qPCR | | |
| mCDKN1A (p21) | CGAGAACGGTGGAACTTTGAC | CCAGGGCTCAGGTAGACCTT |
| mβ-Actin | TACCACCATGTACCCAGGCA | CTCAGGAGGAGCAATGATCTTGAT |
| mXBP1S | GAGTCCGCAGCAGGTG | GTGTCAGAGTCCATGGGA |
| mSPARC | GTGGAAATGGGAGAATTTGAGGA | CTCACACACCTTGCCATGTTT |
| mBLOC1S1 | TCCCGCCTGCTCAAAGAAC | GAGGTGATCCACCAACGCTT |
| mPPP2R1A | GACGGTGACGATTCGCTCTAT | CTGGTCCGTTCAACCCCAAG |
| mRUVBL1 | TTGGGTTGCGGATAAAGGAGA | GGCTGATAGTTTTGCCGTACC |
| mRPL19 | CTGATCAAGGATGGGCTGAT | GCCGCTATGTACAGACACGA |
| dXBP1 | CCTTGGATCTGCCGCAGGGTATAC | AGAGGGCCACAACTTTCCAGAGTGA |
| dSPARC | ATTGAGCGCGAGAAGAGATTGCC | ACTCCGGAATGCACACGCATTT |
| dMYC | TCGCAAAATCTCCTCTTCGG | GGGACACTTGAGCAACTGAA |
| dRPL32 | AGCGCACCAAGCACTTCATA | GTGCGCTTGTTCGATCCGTAA |

* m= Mouse; d= D. melanogaster

**Supplementary Table 2. Cleavage assay. Oligonucleotides sequence used were:**

| Oligonucleotide | Sequence |
|---|---|
| Hppp2r1a-CF1 | AGCCGAATTCTAATACGACTCACTATAGGGAATCTTGGTCGCTA |
| Hppp2r1a-CR840 | TAGCAGGATCCACCATGTAGCGAACACGCCAAGACTT |
| Hppp2r1a-CF1336 | AGCCGAATTCTAATACGACTCACTATAGGGCATTGTGGAGCTGGCTGAGGA |
| Hppp2r1a-CR1919 | TAGCAGGATCCCACCAGAGGCCAGTGTTTGC |
| hRuvbL1-CF1290 | AGCCGAATTCTAATACGACTCACTATAGGGACTCAGTGCAGCTGCTGACC |
| hRuvbL1-CR1650 | TAGCAGGATCCTGTTCTGAAAAGTTTATTTTTA |

**Supplementary Table 3. Fly strains.**

| Experiment | Flies |
|---|---|
| SMART Assays | w*, Nub-GAL4, Ire1-IR; flr$^{3}$/mwh$^{1}$ |
| | w*, Nub-GAL4; flr$^{3}$/mwh$^{1}$ |
| Survival and pPCR Assays | Ire-IR; tub-GAL4 |
| | GFP-IR; tub-GAL4 |
| Immunohistochemistry Analysis | w*, Nub-GAL4; Ire1-IR |
| | w*, Nub-GAL4; GFP-IR |
| MARCM Mosaic Analysis | yw, eyFLP; act<y+<;Gal4, UAS-GFP; FRT82B tubGal80/ Ire1[f02170], FRT82B |
| | yw,ey-FLP;act<y+<Gal4, UAS-GFP; FRT82B tub-Gal80/FRT82B |
